# AP-1 family transcription factors: a diverse family of proteins that regulate varied cellular activities in classical hodgkin lymphoma and ALK+ ALCL

**DOI:** 10.1186/s40164-020-00197-9

**Published:** 2021-01-07

**Authors:** Zuoqiao Wu, Mary Nicoll, Robert J. Ingham

**Affiliations:** 1 Department of Medical Microbiology and Immunology, Li Ka Shing Institute of Virology, University of Alberta, Edmonton, Canada; 2 Present Address: Department of Medicine, University of Toronto, Toronto, Canada; 3 Present Address: Department of Biology, McGill University, Montreal, Canada

**Keywords:** Activator protein-1, Lymphoma, CD30, Hodgkin, ALK+ ALCL

## Abstract

Classical Hodgkin lymphoma (cHL) and anaplastic lymphoma kinase-positive, anaplastic large cell lymphoma (ALK+ ALCL) are B and T cell lymphomas respectively, which express the tumour necrosis factor receptor superfamily member, CD30. Another feature shared by cHL and ALK+ ALCL is the aberrant expression of multiple members of the activator protein-1 (AP-1) family of transcription factors which includes proteins of the Jun, Fos, ATF, and Maf subfamilies. In this review, we highlight the varied roles these proteins play in the pathobiology of these lymphomas including promoting proliferation, suppressing apoptosis, and evading the host immune response. In addition, we discuss factors contributing to the elevated expression of these transcription factors in cHL and ALK+ ALCL. Finally, we examine therapeutic strategies for these lymphomas that exploit AP-1 transcriptional targets or the signalling pathways they regulate.

## Introduction

### AP-1 proteins are a versatile family of dimeric transcription factors

**Fig. 1:**
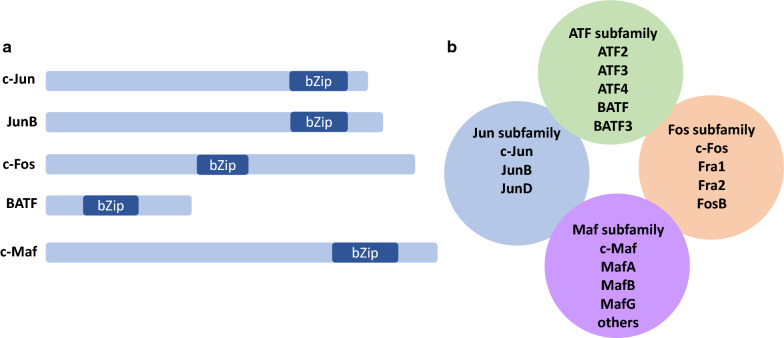
AP-1 proteins are bZip domain-containing transcription factors that comprise four subfamilies.** a** Cartoons illustrating the relative size and location of the basic leucine zipper (bZip) domain in representative AP-1 proteins from each of the different subfamilies. Protein size and the location of the bZip were determined using the Conserved Domain Database [152] (CDD v3.18) at the National Center for Biotechnology Information (NCBI) website. **b** Representative AP-1 family members from the Jun, Fos, ATF, and Maf subfamilies are shown

The activator protein-1 (AP-1) proteins are a collection of transcription factors characterized by the presence of a basic leucine zipper (bZip) domain (Fig. 1a). This family of proteins was first described in the 1980s when *v-Fos* and *v-Jun* were identified as the oncogenic factors associated with FBJ murine osteosarcoma virus [1] and avian sarcoma virus 17 [2], respectively. Subsequent work identified cellular homologues for both genes (c-Fos and c-Jun) [1, 3] as well as related proteins within the Jun (JunB, JunD) and Fos (FRA-1, FRA-2, and FosB) subfamilies [4, 5]. The AP-1 family has further expanded to include members of the ATF and Maf subfamilies [4, 5] (Fig. 1b).

The AP-1 proteins function as dimers. Both homodimers and heterodimers are found; although not all proteins can homodimerize and not all heterodimers are possible [4]. Dimerization is mediated by the leucine zipper (Fig. 1a). This domain adopts an alpha helical structure where leucine side chains interact with the alpha helix of the leucine zipper of the other family member to mediate dimerization [6, 7]. The basic domain is important for interacting with DNA [7]. AP-1 proteins bind 12-*O*-tetradecanoylphorbol-13-acetate (TPA) responsive elements (TRE) (TGA(G/C)TCA), cAMP responsive elements (CRE) (TGACGTCA), and related sequences [8–11]. Individual dimers differ in their DNA binding and transcriptional activities. For example, c-Jun:c-Fos dimers prefer TRE sites, whereas c-Jun:ATF dimers prefer CRE sites [12]. Moreover, c-Jun:c-Fos heterodimers have higher affinity for TRE sites than c-Jun:c-Jun homodimers [8, 9], and dimers containing JunB are less transcriptionally active than those containing c-Jun [13, 14]. While these proteins are primarily thought to function as transcriptional activators, there are situations where they appear to function as repressors ([15–17] as examples). Thus, the AP-1 family is a diverse collection of proteins that generate an even greater collection of dimers with varied DNA binding and transcriptional activities. Not surprisingly, AP-1 family proteins regulate a wide range of cellular and biological activities. These include the cell cycle and proliferation [5, 18], programmed cell death including apoptosis [5, 18, 19] and autophagy [20], and lipid synthesis [21]. As well, AP-1 proteins regulate migration and invasion through modulation of the cytoskeleton [22], and are implicated in inflammatory diseases [23–25], bone development [26–28], the nervous system [29–32], immune cell development and activation [26, 33], and cancer.

### AP-1 proteins are implicated in the development and maintenance of cancers

AP-1 proteins play important roles in multiple malignancies including cancers of the lung [34, 35], breast [25, 36], gastrointestinal tract [25, 37], brain [38–40], skin [41, 42], ovaries [34, 43], and bone [44]. They regulate many of the hallmarks and enabling characteristics of cancer described by Hanahan and Weinberg [45] including sustaining proliferative signalling [5, 18], resisting cell death [5, 18], inducing angiogenesis [46–48], activating invasion and metastasis [22], tumour-promoting inflammation [49], and avoiding immune destruction [33]. AP-1 proteins are also implicated in the pathogenesis of leukemia and lymphoma where these transcription factors can act as oncogenes [50, 51] or tumour suppressors [52–54]. This includes the CD30-positive lymphomas, classical Hodgkin lymphoma (cHL) and anaplastic lymphoma kinase-positive, anaplastic large cell lymphoma (ALK+ ALCL) where AP-1 proteins perform a variety of pro-tumour functions.

### cHL and ALK+ ALCL are CD30-positive lymphomas

The CD30-positive lymphomas are characterized by the expression of tumour necrosis factor receptor superfamily member 8 (TNFRSF8) which is a 120-kDa type I transmembrane glycoprotein more commonly referred to as CD30 [55]. CD30 is recognized by the Ki-1 monoclonal antibody (mAb), first described by Stein and colleagues, which stains the mononuclear Hodgkin cells and multinuclear Reed-Sternberg (HRS) cells of cHL [56]. Subsequently, CD30 was shown to be highly expressed in both ALK+ and ALK- ALCL, as well as a number of other lymphoid cancers and proliferative disorders including cutaneous ALCL, mycosis fungoides, Sézary syndrome, lymphomatoid papulosis, and a subset of diffuse large B cell lymphomas [57–59].

Hodgkin lymphoma, originally called Hodgkin’s disease, was first identified in 1832 by Thomas Hodgkin [60]. Hodgkin lymphoma is a mature B cell lymphoma that is subdivided into classical Hodgkin lymphoma (cHL), and nodular lymphocyte-predominant Hodgkin’s lymphoma (NLPHL) [61]. cHL accounts for approximately 90% of Hodgkin lymphoma cases, and is characterized morphologically by the presence of HRS cells [61]. Interestingly, HRS cells constitute only a small proportion of cells at the tumour site with infiltrating immune cells making up the majority of the tumour mass [62, 63]. The current hypothesis is that HRS cells arise from germinal centre B cells that have failed to undergo apoptosis [62]. HRS cells exhibit the aberrant activation of multiple signalling pathways including the NF-κB [64, 65], JAK/STAT [66–68], and PI3K/Akt [69, 70] pathways.

Anaplastic large cell lymphomas are T cell lymphomas which include ALK+ ALCL, ALK- ALCL, cutaneous ALCL, and breast implant-associated ALCL [58]. In addition to the expression of CD30, ALK+ ALCL are characterized by chromosomal translocations and inversions involving the gene encoding for the *ALK* tyrosine kinase [58]. The most common translocation (~ 80%) is with the gene encoding for *nucleophosmin* (*NPM*) [71]. The resulting t(2;5)(p23;q35) translocation results in a fusion protein (NPM-ALK) consisting of the N-terminal dimerization domain of NPM and the C-terminal kinase and intracellular domains of ALK [71]. This fusion protein exhibits constitutive tyrosine kinase activity and activates many signalling events including the JAK/STAT [72–76] and PI3K/Akt [77, 78] pathways.

**Fig. 2:**
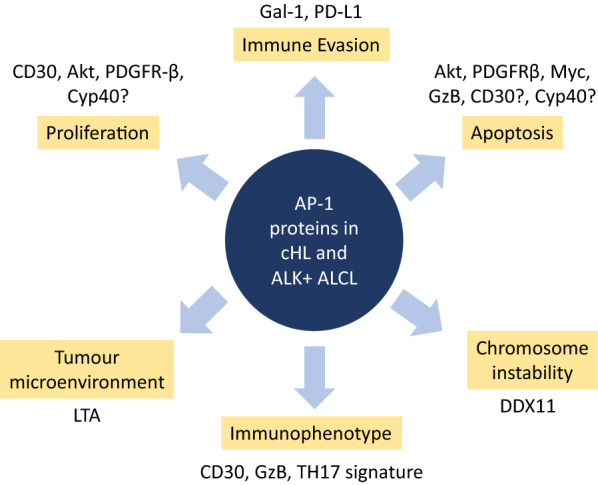
Functions of AP-1 family proteins in cHL and/or ALK+ ALCL. The major cellular activities regulated by AP-1 family proteins, and their transcriptional targets mediating these effects, in cHL and/or ALK+ ALCL are illustrated. Specific details are described in the text

The elevated expression of several AP-1 proteins including c-Jun [79, 80], JunB [79, 81–83], ATF3 [84], BATF [85], and BATF3 [85, 86] has also been described in CD30-positive lymphomas. In the following sub-sections we will discuss how these transcription factors, in collaboration with other signalling pathways, benefit cHL and ALK+ ALCL by promoting proliferation/growth, suppressing apoptosis, and evading the host immune response (Fig. 2). Of note, while many activities/transcriptional targets we discuss have only been described in either ALK+ ALCL or cHL, many could be common to both lymphomas. Likewise, while specific activities/transcriptional targets have been ascribed to particular AP-1 proteins, there may be overlap with other AP-1 family members.

## The function of AP-1 proteins in ALK+ ALCL and cHL

### AP-1 proteins regulate proliferation and growth

Several studies have implicated AP-1 proteins in promoting proliferation in ALK+ ALCL and cHL. Inhibition of AP-1 activity in the L-428 cHL cell line with a dominant negative c-Fos construct, A-Fos, decreased proliferation which was associated with a decrease in cyclin D2 expression [79]. In addition, pharmacological inhibition of the c-Jun activator, c-Jun N-terminal kinase (JNK), in the SU-DHL-1 ALK+ ALCL cell line resulted in cell cycle arrest in G_2_/M phase [87]. This was likely due to the upregulation of the cyclin-dependent kinase (CDK) inhibitor, p21^*cip1*^, and decreased expression of Cyclin A [87]. A similar cell cycle defect was observed in cHL cell lines when JNK was inhibited and this was also associated with an up-regulation of p21^*cip1*^ [88].

A role for specific AP-1 proteins in the regulation of proliferation has been revealed by short interfering RNA (siRNA)/short hairpin RNA (shRNA) knock-down and clustered regularly interspaced short palindromic repeats (CRISPR)/Cas9 knock-out studies. Several groups have reported that JunB knock-down, in most ALK+ ALCL cell lines, decreased proliferation [89–91]. The common cell cycle defect observed in these studies was an increased percentage of cells in G_0_/G_1_ with decreased percentages of cells in G_2_/M [89] or S [90, 91] phase. These defects correlated with decreased expression of CDK2 and multiple cyclins including Cyclin A2, Cyclin D2, Cyclin D3, and Cyclin E, as well as increased expression of CDK inhibitors p14^*ink4A*^, p18^*ink4*^, p21^*cip1*^ and p27^*kip1*^ [90, 91]. In contrast, differing roles for c-Jun in regulating proliferation in ALK+ ALCL have been reported. Two studies observed no effect on proliferation when c-Jun was knocked-down [89, 92], whereas another study found that siRNA-mediated knock-down of c-Jun reduced cell viability and growth which correlated with increased levels of p21^*cip1*^ and decreased levels of Cyclin A and Cyclin D3 [87]. In cHL, stable knock-down of either c-Jun or JunB with shRNA was shown to reduce proliferation. This was characterized by an increased percentage of cells in G_0_/G_1_ and a decreased percentage in S phase, and likely due to elevated p21^*cip1*^ levels [91].

There is also evidence that c-Jun and JunB have overlapping functions with respect to promoting proliferation and/or growth in these lymphomas. For example, in a mouse model of ALK+ ALCL where expression of *NPM-ALK* was driven in T cells by a *CD4* promoter [93], tumour formation was only compromised when both c-Jun and JunB were knocked-out, and double knock-out cells exhibited impaired proliferation [93]. Furthermore, knocking down both c-Jun and JunB with siRNA resulted in a more dramatic reduction in colony formation in Karpas 299 cells compared to single knock-down cells [90].

Other members of the AP-1 family have also been suggested to regulate proliferation in ALK+ ALCL. Schleussner and colleagues demonstrated that CRISPR/Cas9-mediated knock-out of BATF or BATF3 in the Karpas 299 and SUP-M2 cell lines reduced the growth rate of these cells, and reducing the expression of both resulted in an even greater defect [85]. Similar findings were observed in a separate study when BATF was knocked-out of cHL and ALK+ ALCL cell lines [86]. This defect in proliferation is likely due to failure of BATF3, in collaboration with other AP-1 proteins, to promote the expression of c-Myc [86]. Finally, siRNA-mediated knock-down of ATF3 in the L540Cy cHL cell line resulted in decreased [^3^H]-thymidine incorporation consistent with a proliferation defect [84].

Several other AP-1 transcriptional targets have been implicated in the regulation of proliferation and viability. In the CD4-NPM-ALK mouse model, c-Jun and JunB were found to promote transcription of the *Platelet-Derived Growth Factor Receptor β* (*PDGFRβ)*, a receptor tyrosine kinase, which was subsequently found to be highly expressed in ALK+ ALCL patient samples and some ALK+ ALCL cell lines [93]. Importantly, tumour cells isolated from the CD4-NPM-ALK transgenic mice exhibited reduced proliferation when treated with Imatinib, a tyrosine kinase inhibitor which targets PDGFRβ [93]. *CD30* is also a JunB transcriptional target [83, 94, 95]. Knock-down of CD30 was demonstrated to decrease the percentage of cells in S phase and increase the percentage in G_0_/G_1_ in the SU-DHL-1 ALK+ ALCL cell line [90], and found to decrease viability in cHL cell lines [96]. In the former study, CD30 knock-down correlated with an increase in p21^*cip1*^ and p14^*ink4A*^. Likewise, knock-down of the heat shock protein 90 (Hsp90) co-chaperone, Cyclophilin 40 (Cyp40), a JunB transcriptional target in ALK+ ALCL, resulted in reduced viability in multiple ALK+ ALCL cell lines, which could be due in part to a defect in proliferation [97].

Finally, AP-1 proteins also promote proliferation in ALK+ ALCL through PI3K/Akt signalling. The *Akt 1*, *2*, and *3* serine/threonine kinases are transcriptional targets of c-Jun and JunB [98]. Pharmacological inhibition of Akt was shown to decrease the percentage of cells in S phase, and upregulate the CDK inhibitor, p27^*kip1*^ [99]. As well, a dominant negative Akt was shown to affect *in vitro* colony formation and *in vivo* tumour development in BaF3 cells expressing NPM-ALK [78]. Several Akt substrates linked to the regulation of proliferation have been studied in ALK+ ALCL. McDonnell and colleagues demonstrated that NPM-ALK signalling, through PI3K/Akt, leads to phosphorylation and inactivation of the serine/threonine kinase, GSK3β [100]. Inactivation of GSK3β prevented the phosphorylation and degradation of the cell cycle phosphatase, CDC25A [100]. Inactivation of GSK3β in ALK+ ALCL cell lines also promotes proliferation through stabilizing the sonic hedgehog (SHH) pathway transcription factor, Gli1, which results in up-regulation of Cyclin D2 [101]. Akt-mediated activation of mammalian target of rapamycin (mTor) signalling is also important for promoting proliferation, as siRNA-mediated knock-down of mTor decreased the number of cells in S phase [102]. The FOXO3a transcription factor is another substrate of Akt in ALK+ ALCL [103]. Phosphorylation of FOXO3a by Akt prevented FOXO3a from translocating to the nucleus and promoting the transcription of p27^*kip1*^ [103]. Collectively, these studies show that multiple AP-1 proteins, and their transcriptional targets, promote proliferation and growth in cHL and ALK+ ALCL.

### AP-1 proteins and their transcriptional targets influence apoptosis

Protecting cells from apoptosis is also an important function of some AP-1 proteins in cHL and ALK+ ALCL. Forced expression of dominant negative A-Fos in Karpas 299 cells increased the number of cells with condensed or fragmented nuclei, illustrating the importance of AP-1 signalling in protecting these cells from apoptosis [79]. Knock-down of BATF3 with siRNA led to increased Annexin V staining in the SUP-M2 cell line, but not in Karpas 299 cells [85]. Increased apoptosis was also observed in cHL cell lines when ATF3 was knocked-down and this may in part be due to decreased *c-Myc* transcription [84].

Knock-down of all AP-1 proteins is not associated with apoptosis in these lymphomas. Stable knock-down of c-Jun or JunB in multiple cHL and ALK+ ALCL cell lines was not associated with significant apoptosis as measured by TUNEL staining [91]. However, loss of both c-Jun and JunB was associated with increased apoptosis in the CD4-NPM-ALK model as was inhibition of PDGFRβ activity with Imatinib [93]. JunB knock-down in ALK+ ALCL cell lines was found to sensitize cells to etoposide-induced decreases in cell growth and colony formation; however, whether this was due to decreased proliferation and/or increased apoptosis was not explored [90].

There is also evidence that transcriptional targets of AP-1 proteins regulate apoptosis in these lymphomas. As mentioned, knock-down of CD30 [90, 96] or Cyp40 [97] was associated with decreased viability, and a dominant negative Akt decreased colony formation in BaF3 cells expressing NPM-ALK [78]. These phenotypes could be due in part to increased apoptosis. Likewise, knock-down or pharmacological inhibition of Myc was shown to reduce the viability of ALK+ ALCL cell lines [104, 105], and increase the number of sub-G_0_/G_1_ cells [105]. Inhibition of GSK3β by Akt in ALK+ ALCL is also important for preventing the GSK3β-mediated phosphorylation, and subsequent targeting for degradation, of the pro-survival Bcl-2 family member, Mcl-1 [100]. This same study found that inhibition of GSK3β decreased poly(ADP-ribose) polymerase (PARP) cleavage in cells treated with an ALK inhibitor [100]. Other Akt substrates including mTor [102] and FOXO3a [103] are also important for promoting survival in ALK+ ALCL, and inhibition of SHH/GLI1 signalling in ALK+ ALCL cell lines resulted in an increased percentage of Annexin V-positive cells [101].

On the other hand, there is evidence that AP-1 transcriptional targets may promote apoptosis in ALK+ ALCL. The serine protease, Granzyme B (GzB), is highly expressed in ALK+ ALCL [106, 107], and its transcription is promoted by NPM-ALK signalling and JunB [108]. GzB is primarily expressed by cytotoxic T lymphocytes (CTLs) and natural killer (NK) cells, where it allows these cells to kill virally-infected or transformed cells [109]. While knock-down of GzB in ALK+ ALCL cell lines did not result in appreciable apoptosis on its own, knock-down cells were less sensitive to staurosporine and doxorubicin-induced apoptosis [110]. This suggests that GzB expression could be one reason why ALK+ ALCL patients are generally responsive to chemotherapy.

### AP-1 proteins regulate immunomodulatory genes

There are several immunomodulatory molecules which are AP-1 transcription targets in cHL and ALK+ ALCL. Galectin-1 (Gal-1) is an immunoglycan highly expressed in cHL and ALK+ ALCL patients and its expression strongly correlates with c-Jun levels [111–113]. Moreover, an AP-1 site within the *Gal-1* enhancer was shown to bind c-Jun and promote *Gal-1* transcription [111]. In cHL, Gal-1 was shown to create an immunosuppressive tumour microenvironment by promoting the expression of T helper 2 (T_H_2)-promoting cytokines and increasing the number of regulatory T cells (Tregs) [111]. In addition, analysis of patient samples demonstrated that HRS cells with high Gal-1 expression had lower infiltrating CD8-positive T cells, and *in vitro* experiments with recombinant Gal-1 demonstrated that Gal-1 can impair CD8 proliferation and effector function [113].

The transcription of *Program death-ligand 1* (*PD-L1*) is also mediated by AP-1 transcription factors in cHL and ALK+ ALCL [114, 115]. PD-L1 is a ligand for the immune inhibitory receptor, program death-1 (PD-1), and engagement of PD-1 by PD-L1 allows cancers expressing PD-L1 to evade killing by CTLs and NK cells [116, 117]. Both c-Jun and JunB bind to a *PD-L1* enhancer region, and this was found to be important for promoting *PD-L1* transcription [115]. Furthermore, BATF3, together with the IRF4 transcription factor, was found to be important for PD-L1 expression in ALK+ ALCL [114], and inhibition of the PD-1/PD-L1 signalling axis in ALK+ ALCL cell lines was found to increase the ability of these cell lines to activate T cells and be killed by NK cells [114].

### Additional activities

There are also additional functions performed by the AP-1 proteins in cHL and ALK+ ALCL. JunB has been linked to genomic instability in ALK+ ALCL through directly repressing the expression of the DEAD-box helicase, DDX11, which regulates sister chromatid cohesion [118]. Finally, c-Jun and c-Fos activity were implicated in the expression of Lymphotoxin-α (LTA) in cHL [119]. LTA is a member of the TNF family and is important for lymphoid organ development, inflammation, and antiviral responses [120, 121]. LTA secreted by cHL cell lines facilitated the interaction of CD4+ T cells with human umbilical vein endothelial cells (HUVECs) through the induction of the ICAM-1, VCAM-1, and E-selectin adhesion molecules in HUVECs [119]. Thus, through secreting LTA, HRS cells may contribute to the immune cell infiltrate characteristic of cHL.

AP-1 proteins are also critical for the expression of genes that characterize ALK+ ALCL and cHL. This includes CD30 [83, 94, 95] and GzB, with the latter being a hallmark of the cytotoxic phenotype of ALK+ ALCL [106, 107]. BATF/BATF3 are important for expression of genes in ALK+ ALCL that are associated with the T_H_17/group 3 innate lymphoid cell gene signature observed in this lymphoma [85]. Likewise, BATF3 expression is important for both the expression and repression of genes that characterize cHL [122].

## **Multiple mechanisms account for elevated AP-1 protein expression**

In the previous section we discussed the many important activities influenced by AP-1 proteins in cHL and ALK+ ALCL. In this section we will examine the mechanisms and signalling events that lead to their elevated expression and/or activation in these lymphomas.

Atsaves et al. reported that *JunB* gene amplifications are common in ALK+ ALCL, but this did not correlate with increased JunB expression [90]. *JunB* transcription in ALK+ ALCL is dependent on NPM-ALK [123, 124], and on signalling events initiated by CD30 through a Mek/Erk/Ets-1 pathway [89, 94, 124]. The latter pathway is also important for promoting *JunB* transcription in cHL [94, 124], and NF-κB has also been suggested to increase *JunB* transcription [79], though others have reported different findings [94]. Importantly, the fact that CD30 is both a target and regulator of JunB means that a positive feedback loop is generated that ensures high levels of CD30 and JunB in these lymphomas. Levels of BATF3 in ALK+ ALCL are regulated by NPM-ALK/STAT3 signalling [114], and JAK/STAT signalling is also important for BATF3 expression in cHL [86]. This highlights an example of cross-talk between the AP-1 and JAK/STAT signalling pathways in these lymphomas. Signalling through Sphingosine 1-phosphate receptor 1 (S1PR1) also activates BATF3 transcription in cHL through a PI3K-dependent pathway, and BATF further promotes S1PR1 transcription generating a positive feedback loop [122]. As well, the IRF5 transcription factor promotes the transcription of multiple AP-1 genes in cHL including *c-Jun*, *JunB*, and *ATF3*; however, whether this up-regulation was directly or indirectly mediated by IRF5 was not determined [125].

Post-transcriptional and post-translational mechanisms also influence c-Jun/JunB levels and activity. Recently, expression of miR-939 in ALK+ ALCL was found to reduce JunB levels [126], and JunB translation was promoted in ALK+ ALCL by targeting *JunB* mRNA to polysomes via a PI3K/Akt/mTor-dependent pathway [89]. There are several examples of the post-translational regulation of the AP-1 proteins in cHL and ALK+ ALCL. JNK, activated by NPM-ALK signalling, mediates phosphorylation and activation of c-Jun in ALK+ ALCL [87]. As well, a lack of GSK3β activity in this lymphoma has been argued to lead to a failure of JunB to be phosphorylated, and subsequently targeted for degradation, by the Fbxw7 E3 ubiquitin ligase [127]. Because Akt is a transcriptional target of JunB, and signalling mediated by Akt regulates JunB translation and stability, this illustrates cross-talk between these two pathways in ALK+ ALCL. Loss of expression of another E3 ubiquitin ligase, PDLIM2, was found to be common to both cHL and ALK+ ALCL, and reduced PDLIM2 expression increased AP-1 transcriptional activity through an unknown mechanism [128]. Thus, many factors contribute to the elevated expression of AP-1 family proteins in cHL and ALK+ ALCL.

## Therapies directed at targets of AP-1 proteins in cHL and ALK+ ALCL

### Frontline treatments

**Fig. 3:**
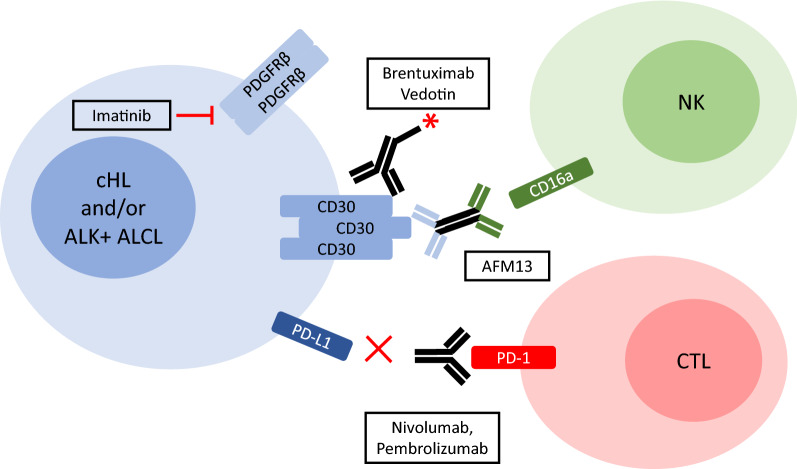
Therapies targeting AP-1 regulated genes in cHL and/or ALK+ ALCL. Therapies targeting the protein products of AP-1 transcriptional targets in cHL and/or ALK+ ALCL are highlighted. These include the small molecule tyrosine kinase inhibitor, Imatinib, which targets PDGFRβ, blocking antibodies that interfere with PD-1/PD-L1 interaction (Nivolumab, Pembrolizumab), a bispecfic antibody that recruits NK cells to cells expressing CD30 (AFM13), and an antibody-toxin conjugate which targets CD30-positive cells (Brentuximab Vedotin). Additional details are provided in the text

Frontline treatments for ALK+ ALCL are combination chemotherapy regimens. These are generally quite effective at treating the disease with event free survival ranging from 68 to 76% depending on the trial [129]. cHL is also treated successfully with combination chemotherapy in conjunction with radiotherapy [130]. Despite the success of these treatments, some patients are refractory to treatment and others relapse [129, 130]. Also, there are long-term negative consequences associated with chemotherapy and radiation [131]. Thus, precision medicine approaches that exploit specific features of these cancers have been developed. This includes ALK tyrosine kinase inhibitors, such as Crizotinib, which are currently in clinical trials to treat ALK+ ALCL [129]. Pertinent to this review, there are several therapies directed at AP-1 transcriptional targets (Fig. 3). For example, inhibition of PDGFRβ kinase activity with Imatinib was shown to successfully treat an ALK+ ALCL patient that was refractory to chemotherapy and had relapsed after autologous stem cell transplantation [93]. In addition, several therapies targeting CD30 or the interaction between PD-1/PD-L1 have been developed and we will discuss these in more detail in the next sub-section.

### Therapies targeting CD30 and PD-1/PD-L1

CD30 therapies include several monoclonal antibodies as well as antibody conjugates [55]. Brentuximab vedotin (also known as SGN-35) is a CD30-specific monoclonal antibody (mAb) conjugated to the anti-mitotic agent, monomethylauristatin E (MMAE) via a cathepsin protease cleavable linker [132]. The anti-CD30 Ab component of brentuximab vedotin binds to CD30, which leads to the internalization of the Ab-conjugate, cleavage of the peptide linker, and release of MMAE. MMAE is a synthetic compound, related to a compound isolated from a shell-less marine mollusk, which kills cells through inhibiting tubulin polymerization [133]. Brentiximab vedotin is effective in treating relapsed or refractory cHL and ALK+ ALCL [134–136], and shown to be promising as a frontline treatment for these lymphomas in combination with chemotherapy [137, 138].

Other treatments targeting CD30 include bispecific antibodies such as AFM13. AFM13 consists of the heavy and light chain variable regions from the HRS-3 anti-CD30 mAb fused as a single polypeptide to the heavy and light chain variable regions of a mAb that recognizes CD16a expressed on NK cells [139]. This polypeptide dimerizes to form a tetravalent molecule with two CD30 and two CD16a binding sites, and mediates killing of cHL and ALK+ ALCL cell lines by recruiting NK cells [139, 140]. Finally, chimeric antigen receptor (CAR) T cells that specifically target CD30 are also being investigated for the treatment of CD30-positive lymphomas [141].

Interfering with the interaction between PD-L1-expressing tumour cells and T cells expressing PD-1 is an effective therapy for many cancers [116], and this includes cHL and ALK+ ALCL [142]. The PD-1 binding mAbs, Nivolumab [143, 144] and Pembrolizumab [145, 146] have been shown to be effective in treating relapsed or resistant cHL. Furthermore, two cases reports have reported a positive effect of Nivolumab treatment on individual ALK+ ALCL patients that failed other treatments [147, 148].

## Concluding remarks

In this review, we discussed the key roles AP-1 proteins play in the pathobiology of cHL and ALK+ ALCL, the events that lead to the aberrant expression of these proteins, and how AP-1 transcriptional targets, or the components of pathways they function within, can be exploited as therapeutic targets. Nonetheless, there are still important questions to be addressed.

We need to know more mechanistically how AP-1 proteins regulate pro-tumour functions in these lymphomas. For example, studies have demonstrated the knock-down of AP-1 proteins results in the down-regulation of cyclins and CDKs and the up-regulation of CDK inhibitors. However, in most cases whether these changes are a direct transcriptional consequence of reduced expression of the AP-1 protein, or more likely, indirect due to dysregulation of signalling events mediated by transcriptional targets is not clear. Moreover, with regard to the latter possibility, determining which transcriptional targets are the most critical, and how they signal to regulate proliferation, or other activities, requires further investigation.

Proteomic and microarray studies have been performed to identify genome-wide dysregulated genes in knock-down cells for some family members [86, 97, 108, 122]. Extending these experiments to additional AP-1 proteins, and although technically more challenging, when multiple AP-1 proteins are knocked-down or knocked-out will provide a more comprehensive understanding of cellular activities regulated by these transcription factors. Likewise, chromatin immunoprecipitation-sequencing (ChIP-Seq) experiments will complement these studies by globally characterizing genomic sites occupied by these transcription factors and identifying those genes more likely to be direct transcriptional targets. In addition, ChIP-Seq experiments will help reveal which genes are more likely regulated by individual AP-1 proteins versus those regulated by multiple family members. Likewise, although many AP-1 proteins are aberrantly expressed in these lymphomas, little is known about the abundance and function of specific AP-1 dimers. Quantitative mass spectrometry studies and experiments utilizing defined AP-1 dimers [149] will help address these questions.

Finally, as more AP-1-regulated genes are identified, some of these, or the signalling pathways they function within, could become novel treatments. Even the AP-1 family proteins themselves could become drug targets. Small molecules and peptides that interfere with AP-1 DNA binding or dimer formation are being investigated as therapeutics [150, 151] and these could be attractive treatments for cHL and ALK+ ALCL.

## Data Availability

Data sharing is not applicable to this article as no datasets were generated or analyzed during the current study.

## Abbreviations

cHL: Classical Hodgkin lymphoma
ALK+ ALCL: Anaplastic lymphoma kinase positive, anaplastic large cell lymphoma
AP-1: Activator protein-1
bZip: basic leucine zipper
TPA: 12-*O*-tetradecanoylphorbol-13-acetate
TRE: 12-*O*-tetradecanoylphorbol-13-acetate responsive elements
CRE: cAMP responsive elements
TNFRSF8: Tumour necrosis factor receptor superfamily member 8
NLPHL: Nodular lymphocyte predominant Hodgkin lymphoma
HRS: Hodgkin and Reed-Sternberg cells
NPM: Nucleophosmin
S1P: Sphingosine-1-phosphate
JNK: c-Jun N-terminal kinase
CDK: Cyclin-dependent kinase
PDGFRβ: Platelet-derived growth factor β
HSP90: Heat shock protein 90
Cyp40: Cyclophilin 40
SHH: SONIC hedgehog
mTOR: mammalian target of rapamycin
PARP: Poly(ADP-ribose) polymerase
GzB: Granzyme B
CTLs: Cytotoxic T lymphocytes
NK: Natural killer
Gal-1: Galectin-1
TH2: T-helper 2
Tregs: Regulatory T cells
PD-L1: Programmed death ligand-1
PD-1: Programmed death-1
LTA: Lymphotoxin-α
HUVECs: Human umbilical vein endothelial cells
EFS: Event free survival
mAb: Monoclonal antibody
MMAE: Monomethylauristatin E
CAR: Chimeric antigen receptor
ChIP-seq: Chromatin immunoprecipitation sequencing
